# Supplementary material for: Mapping the evolution of fertility support policies in China: A content and instrumental analysis
Source: PLoS One. 2025 Oct 9;20(10):e0332137. doi: 10.1371/journal.pone.0332137 (PMC12510515; doi:10.1371/journal.pone.0332137)
Supplement: S1 Appendix — (ZIP) [file pone.0332137.s001.zip › S1 Appendix. 226 original policy documents/138-中共中央、国务院关于优化生育政策促进人口长期均衡发展的决定(FBM-CLI-16-5016842).doc]

中共中央、国务院关于优化生育政策促进人口长期均衡发展的决定

**发布部门：** [中国共产党中央委员会](https://www.pkulaw.com/law/chl?Aggs.IssueDepartment=a01) [国务院](https://www.pkulaw.com/law/chl?Aggs.IssueDepartment=5)

**发布日期：**2021.06.26

**实施日期：**2021.06.26

**时效性：** [现行有效](https://www.pkulaw.com/law/chl?Aggs.TimelinessDic=01)

**效力级别：** [党内法规制度](https://www.pkulaw.com/law/chl?Aggs.EffectivenessDic=XR12)

**法规类别：** [计划生育管理](https://www.pkulaw.com/law/chl?Aggs.Category=09705)

中共中央、国务院关于优化生育政策促进人口长期均衡发展的决定
（2021年6月26日）

　　人口发展是关系中华民族发展的大事情。为贯彻落实党的十九大和十九届二中、三中、四中、五中全会精神，促进人口长期均衡发展，现就优化生育政策，实施一对夫妻可以生育三个子女政策，并取消社会抚养费等制约措施、清理和废止相关处罚规定，配套实施积极生育支持措施（以下简称实施三孩生育政策及配套支持措施），作出如下决定。
　　一、充分认识优化生育政策、促进人口长期均衡发展的重大意义
　　党和国家始终坚持人口与发展综合决策，科学把握人口发展规律，坚持计划生育基本国策，有力促进了经济发展和社会进步，为全面建成小康社会奠定了坚实基础。党的十八大以来，党中央高度重视人口问题，根据我国人口发展变化形势，作出逐步调整完善生育政策、促进人口长期均衡发展的重大决策，各项工作取得显著成效。当前，进一步适应人口形势新变化和推动高质量发展新要求，实施三孩生育政策及配套支持措施，具有重大意义。
　　（一）有利于改善人口结构，落实积极应对人口老龄化国家战略。老龄化是全球性人口发展大趋势，也是我国发展面临的重大挑战。预计“十四五”期间我国人口将进入中度老龄化阶段，2035年前后进入重度老龄化阶段，将对经济运行全领域、社会建设各环节、社会文化多方面产生深远影响。实施三孩生育政策及配套支持措施，有利于释放生育潜能，减缓人口老龄化进程，促进代际和谐，增强社会整体活力。

　　（二）有利于保持人力资源禀赋优势，应对世界百年未有之大变局。人口是社会发展的主体，也是影响经济可持续发展的关键变量。实施三孩生育政策及配套支持措施，有利于未来保持适度人口总量和劳动力规模，更好发挥人口因素的基础性、全局性、战略性作用，为高质量发展提供有效人力资本支撑和内需支撑。

　　（三）有利于平缓总和生育率下降趋势，推动实现适度生育水平。群众生育观念已总体转向少生优育，经济负担、子女照料、女性对职业发展的担忧等成为制约生育的主要因素。实施三孩生育政策及配套支持措施，促进生育政策与相关经济社会政策同向发力，有利于满足更多家庭的生育意愿，有利于提振生育水平。

　　（四）有利于巩固全面建成小康社会成果，促进人与自然和谐共生。今后一个时期，我国人口众多的基本国情不会改变，人口与资源环境承载力仍然处于紧平衡状态，脱贫地区以及一些生态脆弱、资源匮乏地区人口与发展矛盾仍然比较突出。实施三孩生育政策及配套支持措施，有利于进一步巩固脱贫攻坚和全面建成小康社会成果，引导人口区域合理分布，促进人口与经济、社会、资源、环境协调可持续发展。
　　二、指导思想、主要原则和目标

　　（五）指导思想。坚持以习近平新时代中国特色社会主义思想为指导，立足新发展阶段、贯彻新发展理念、构建新发展格局，实施积极应对人口老龄化国家战略，实施三孩生育政策及配套支持措施，改革服务管理制度，提升家庭发展能力，推动实现适度生育水平，促进人口长期均衡发展，为建设富强民主文明和谐美丽的社会主义现代化强国、实现中华民族伟大复兴的中国梦提供坚实基础和持久动力。

　　（六）主要原则
　　--以人民为中心。顺应人民群众期盼，积极稳妥推进优化生育政策，促进生育政策协调公平，满足群众多元化的生育需求，将婚嫁、生育、养育、教育一体考虑，切实解决群众后顾之忧，释放生育潜能，促进家庭和谐幸福。
　　--以均衡为主线。把促进人口长期均衡发展摆在全党全国工作大局、现代化建设全局中谋划部署，兼顾多重政策目标，统筹考虑人口数量、素质、结构、分布等问题，促进人口与经济、社会、资源、环境协调可持续发展，促进人的全面发展。
　　--以改革为动力。着眼于我国人口发展面临的突出矛盾和问题，着眼于现代化建设战略安排，深化改革，破除影响人口长期均衡发展的思想观念、政策法规、体制机制等制约因素，提高人口治理能力和水平。
　　--以法治为保障。坚持重大改革于法有据、依法实施，将长期以来党领导人民在统筹解决人口问题方面的创新理念、改革成果、实践经验转化为法律，保障人民群众合法权益，保障新时代人口工作行稳致远，保障人口发展战略目标顺利实现。

　　（七）主要目标
　　到2025年，积极生育支持政策体系基本建立，服务管理制度基本完备，优生优育服务水平明显提高，普惠托育服务体系加快建设，生育、养育、教育成本显著降低，生育水平适当提高，出生人口性别比趋于正常，人口结构逐步优化，人口素质进一步提升。
　　到2035年，促进人口长期均衡发展的政策法规体系更加完善，服务管理机制运转高效，生育水平更加适度，人口结构进一步改善。优生优育、幼有所育服务水平与人民群众对美好生活的需要相适应，家庭发展能力明显提高，人的全面发展取得更为明显的实质性进展。
　　三、组织实施好三孩生育政策

　　（八）依法实施三孩生育政策。修改《[中华人民共和国人口与计划生育法](https://www.pkulaw.com/chl/18c6c388fc14eaa9bdfb.html)》，提倡适龄婚育、优生优育，实施三孩生育政策。各省（自治区、直辖市）综合考虑本地区人口发展形势、工作基础和政策实施风险，做好政策衔接，依法组织实施。

　　（九）取消社会抚养费等制约措施。取消社会抚养费，清理和废止相关处罚规定。将入户、入学、入职等与个人生育情况全面脱钩。依法依规妥善处理历史遗留问题。对人口发展与经济、社会、资源、环境矛盾较为突出的地区，加强宣传倡导，促进相关惠民政策与生育政策有效衔接，精准做好各项管理服务。

　　（十）建立健全人口服务体系。以“一老一小”为重点，建立健全覆盖全生命周期的人口服务体系。加强基层服务管理体系和能力建设，增强抚幼养老功能。落实生育登记制度，做好生育咨询指导。推进出生医学证明、儿童预防接种、户口登记、医保参保、社保卡申领等“出生一件事”联办。

　　（十一）加强人口监测和形势研判。完善国家生命登记管理制度，健全覆盖全人群、全生命周期的人口监测体系，密切监测生育形势和人口变动趋势。依托国家人口基础信息库等平台，实现教育、公安、民政、卫生健康、医保、社保等人口服务基础信息融合共享、动态更新。建立人口长期均衡发展指标体系，健全人口预测预警制度。
　　四、提高优生优育服务水平

　　（十二）保障孕产妇和儿童健康。全面落实妊娠风险筛查与评估、高危孕产妇专案管理、危急重症救治、孕产妇死亡个案报告和约谈通报等母婴安全五项制度。实施妇幼健康保障工程，加快推进各级妇幼保健机构标准化建设和规范化管理，加强危重孕产妇、新生儿救治能力及儿科建设，夯实县乡村三级基层网络，加快补齐生育相关公共服务短板。促进生殖健康服务融入妇女健康管理全过程。加强儿童保健门诊标准化、规范化建设，加强对儿童青少年近视、营养不均衡、龋齿等风险因素和疾病的筛查、诊断、干预。做好儿童基本医疗保障工作。

　　（十三）综合防治出生缺陷。健全出生缺陷防治网络，落实三级预防措施。加强相关知识普及和出生缺陷防控咨询，强化婚前保健，推进孕前优生健康检查，加强产前筛查和诊断，推动围孕期、产前产后一体化管理服务和多学科协作。扩大新生儿疾病筛查病种范围，促进早筛早诊早治。做好出生缺陷患儿基本医疗和康复救助工作。

　　（十四）规范人类辅助生殖技术应用。强化规划引领，严格技术审批，建设供需平衡、布局合理的人类辅助生殖技术服务体系。加强人类辅助生殖技术服务监管，严格规范相关技术应用。开展孕育能力提升专项攻关，规范不孕不育诊治服务。
　　五、发展普惠托育服务体系

　　（十五）建立健全支持政策和标准规范体系。将婴幼儿照护服务纳入经济社会发展规划，强化政策引导，通过完善土地、住房、财政、金融、人才等支持政策，引导社会力量积极参与。以市地级行政区为单位制定整体解决方案，建立工作机制，推进托育服务健康发展。加大专业人才培养力度，依法逐步实行从业人员职业资格准入制度。发展智慧托育等新业态，培育托育服务、乳粉奶业、动画设计和制作等行业民族品牌。

　　（十六）大力发展多种形式的普惠服务。发挥中央预算内投资的引导和撬动作用，推动建设一批方便可及、价格可接受、质量有保障的托育服务机构。支持有条件的用人单位为职工提供托育服务。鼓励国有企业等主体积极参与各级政府推动的普惠托育服务体系建设。加强社区托育服务设施建设，完善居住社区婴幼儿活动场所和服务设施。制定家庭托育点管理办法。支持隔代照料、家庭互助等照护模式。支持家政企业扩大育儿服务。鼓励和支持有条件的幼儿园招收2至3岁幼儿。

　　（十七）加强综合监管。各类机构开展婴幼儿照护服务必须符合国家和地方相关标准和规范，并对婴幼儿安全和健康负主体责任。地方政府要承担监管责任，建立健全登记备案制度、信息公示制度、评估制度，加强动态管理，建立机构关停等特殊情况应急处置机制。
　　六、降低生育、养育、教育成本

　　（十八）完善生育休假与生育保险制度。严格落实产假、哺乳假等制度。支持有条件的地方开展父母育儿假试点，健全假期用工成本分担机制。继续做好生育保险对参保女职工生育医疗费用、生育津贴待遇等的保障，做好城乡居民医保参保人生育医疗费用保障，减轻生育医疗费用负担。

　　（十九）加强税收、住房等支持政策。结合下一步修改[个人所得税法](https://www.pkulaw.com/chl/d74fa423af05cffdbdfb.html)，研究推动将3岁以下婴幼儿照护费用纳入个人所得税专项附加扣除。地方政府在配租公租房时，对符合当地住房保障条件且有未成年子女的家庭，可根据未成年子女数量在户型选择等方面给予适当照顾。地方政府可以研究制定根据养育未成年子女负担情况实施差异化租赁和购买房屋的优惠政策。

　　（二十）推进教育公平与优质教育资源供给。推进城镇小区配套幼儿园治理，持续提升普惠性幼儿园覆盖率，适当延长在园时长或提供托管服务。推进义务教育优质均衡发展和城乡一体化，有效解决“择校热”难题。依托学校教育资源，以公益普惠为原则，全面开展课后文体活动、社会实践项目和托管服务，推动放学时间与父母下班时间衔接。改进校内教学质量和教育评价，将学生参加课外培训频次、费用等情况纳入教育督导体系。平衡家庭和学校教育负担，严格规范校外培训。

　　（二十一）保障女性就业合法权益。规范机关、企事业等用人单位招录、招聘行为，促进妇女平等就业。落实好《[女职工劳动保护特别规定](https://www.pkulaw.com/chl/cdf82292dbb62ee9bdfb.html)》，定期开展女职工生育权益保障专项督查。为因生育中断就业的女性提供再就业培训公共服务。将生育友好作为用人单位承担社会责任的重要方面，鼓励用人单位制定有利于职工平衡工作和家庭关系的措施，依法协商确定有利于照顾婴幼儿的灵活休假和弹性工作方式。适时对现行有关休假和工作时间的政策规定进行相应修改完善。
　　七、加强政策调整有序衔接

　　（二十二）维护好计划生育家庭合法权益。对全面两孩政策调整前的独生子女家庭和农村计划生育双女家庭，继续实行现行各项奖励扶助制度和优惠政策。探索设立独生子女父母护理假制度。加强立法，保障响应党和国家号召、实行计划生育家庭的合法权益。

　　（二十三）建立健全计划生育特殊家庭全方位帮扶保障制度。根据经济社会发展水平等因素，实行特别扶助制度扶助标准动态调整。对符合条件的计划生育特殊家庭成员，落实基本养老、基本医疗保障相关政策；优先安排入住公办养老机构，提供无偿或低收费托养服务；对住房困难的，优先纳入住房保障。有条件的地方可对计划生育特殊家庭成员中的生活长期不能自理、经济困难的老年人发放护理补贴。落实好扶助所需资金，有条件的地方可探索建立公益金或基金，重点用于帮扶计划生育特殊家庭。

　　（二十四）建立健全政府主导、社会组织参与的扶助关怀工作机制。通过公开招投标方式，支持有资质的社会组织接受计划生育特殊家庭委托，开展生活照料、精神慰藉等服务，依法代办入住养老机构、就医陪护等事务。深入开展“暖心行动”。建立定期巡访制度，落实计划生育特殊家庭“双岗”联系人制度，扎牢织密帮扶安全网。
　　八、强化组织实施保障

　　（二十五）加强党的领导。各级党委和政府要提高政治站位，增强国情、国策意识，坚持一把手亲自抓、负总责，坚持和完善目标管理责任制，加强统筹规划、政策协调和工作落实，推动出台积极生育支持措施，确保责任到位、措施到位、投入到位、落实到位。

　　（二十六）动员社会力量。加强政府和社会协同治理，充分发挥工会、共青团、妇联等群团组织在促进人口发展、家庭建设、生育支持等方面的重要作用。积极发挥计划生育协会作用，加强基层能力建设，做好宣传教育、生殖健康咨询服务、优生优育指导、计划生育家庭帮扶、权益维护、家庭健康促进等工作。鼓励社会组织开展健康知识普及、婴幼儿照护服务等公益活动。以满足老年人生活需求和营造婴幼儿健康成长环境为导向，开展活力发展城市创建活动。

　　（二十七）深化战略研究。面向建设社会主义现代化强国和实现中华民族伟大复兴，持续深化国家人口中长期发展战略和区域人口发展规划研究，完善人口空间布局，优化人力资源配置。加强新时代中国特色人口学科和理论体系建设，发展人口研究高端智库，促进国际交流合作。

　　（二十八）做好宣传引导。加强政策宣传解读，把各地区各部门和全社会的思想行动统一到党中央重大决策部署上来，引导社会各界正确认识人口的结构性变化，弘扬主旋律、汇聚正能量，及时妥善回应社会关切，营造良好氛围。弘扬中华民族传统美德，尊重生育的社会价值，提倡适龄婚育、优生优育，鼓励夫妻共担育儿责任，破除高价彩礼等陈规陋习，构建新型婚育文化。

　　（二十九）加强工作督导。各省（自治区、直辖市）要按照本决定要求，制定实施方案，狠抓任务落实，及时研究解决苗头性、倾向性问题，确保优化生育政策取得积极成效。各省（自治区、直辖市）党委和政府每年要向党中央、国务院报告本地区人口工作情况，中央将适时开展督查。

（新华社北京2021年7月20日电）

© 北大法宝：（[www.pkulaw.com](https://www.pkulaw.com/)) 专业提供法律信息、法学知识和法律软件领域各类解决方案。北大法宝为您提供丰富的参考资料，正式引用法规条文时请与标准文本核对。
欢迎查看所有[产品和服务](https://www.pkulaw.com/helps/2.html)。法宝快讯：[如何快速找到您需要的检索结果？](https://www.pkulaw.com/helps/70.html)[法宝](https://www.pkulaw.com/helps/69.html)V6有何新特色？

扫描二维码阅读原文

**原文链接：**<https://www.pkulaw.com/chl/1247bc81dccaeed3bdfb.html>
